# Elbow Dislocation With Associated Essex-Lopresti Injury: A Case Treated Conservatively

**DOI:** 10.7759/cureus.32099

**Published:** 2022-12-01

**Authors:** Vasileios Giannatos, Panagiotis Antzoulas, Harry Charalampus, Vasileios Athanasiou, Andreas Panagopoulos, Zinon Kokkalis

**Affiliations:** 1 Department of Orthopedics, University Hospital of Patras, Patras, GRC

**Keywords:** forearm instability, interosseus membrane, iom, eli, ell, lopresti, essex, essex-lopresti

## Abstract

A 23-year-old woman suffered a posterior elbow dislocation, distal radius intraarticular fracture, distal radioulnar joint subluxation, and coronoid process fracture, suggesting an Essex-Lopresti injury variant. Closed reduction for the elbow dislocation was performed, and the limb was immobilized at a 90-degree angle with the forearm in a neutral position with a long posterior splint. Three months later complete fracture healing was noted radiologically. One year post-injury full range of motion regarding flexion, pronation, and supination was achieved with only 10 degrees of extension deficit remaining, suggesting a case of Essex-Lopresti injury managed conservatively with excellent results.

## Introduction

The Essex-Lopresti injury consists of a radial head fracture, inter-osseus membrane (IOM) disruption, and distal radio-ulnar joint (DRUJ) dislocation [1]. The forearm ring consists of the radius, ulna, DRUJ, and proximal radio-ulnar joint (PRUJ) and contributes to the distribution of forces along the ring. The annular ligament, triangular fibrocartilage complex (TFCC), and IOM contribute to the ring stabilization in the PRUJ, DRUJ, and middle radio-ulnar joint (MRUJ), creating two anatomical and one functional locker [2, 3]. Disruption of two or more components of the ring can destabilize the forearm. Recognized patterns of ring disruption consist of Galeazzi, Monteggia, and Essex-Lopresti fractures and are less frequent than isolated forearm fractures, however, their consequences can be devastating. In this case report, we are presenting the case of an Essex-Lopresti injury (ELI). The Essex-Lopresti is an unusual injury with an incidence of 1% of all radial head fractures [2]. It is the most severe of the eponymous ring disruption patterns as it is characterized as a three-locker injury, according to the locker-based classification. More specifically, Essex-Lopresti consists of a radial head fracture along with a concomitant DRUJ dislocation and IOM disruption, although variants do exist. The first confirmed case was recorded by Curr and Coe in 1946 [4]. Later, in 1951 Essex-Lopresti described another two cases, providing useful insight for the management that constitutes the core of treatment until today [1]. The mechanism of injury is usually a high-energy fall on an outstretched supinated hand, leading to longitudinal energy transmission from the hand to the wrist, forearm, and elbow [5].

## Case presentation

**Figure 1:**
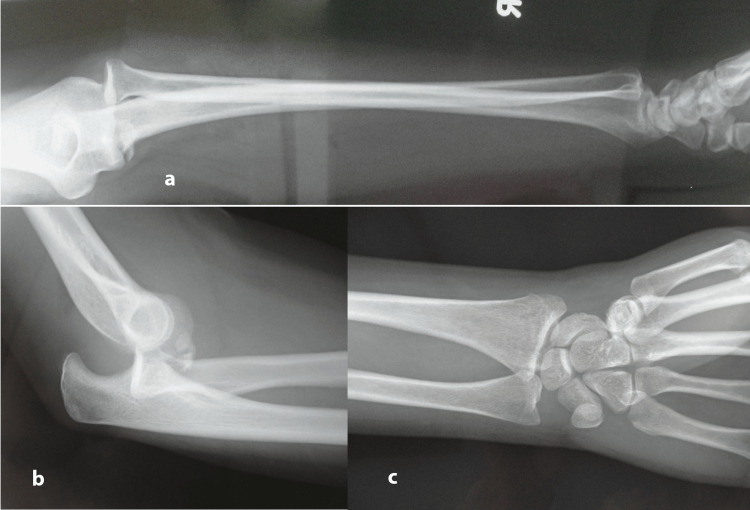
Anteroposterior, (a) lateral elbow, (b) anteroposterior wrist, (c) X-rays at time of injury. Posterior elbow dislocation along with DRUJ dislocation are evident. DRUJ: distal radio-ulnar joint

**Figure 2:**
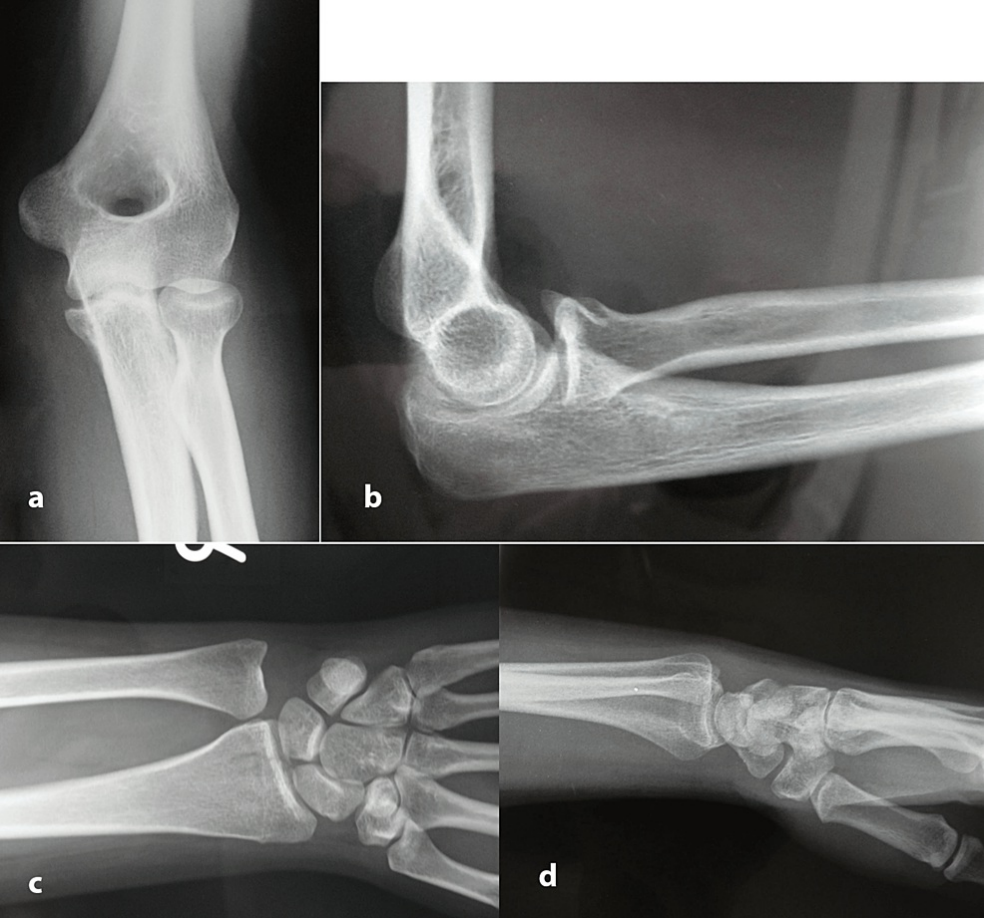
Anteroposterior (a) and lateral elbow (b) and anteroposterior (c) and lateral wrist (d) x-rays after reduction. The coronoid process fracture (type 2) and the intraarticular distal radius fracture are evident. No positive ulnar variance is noted.

**Figure 3:**
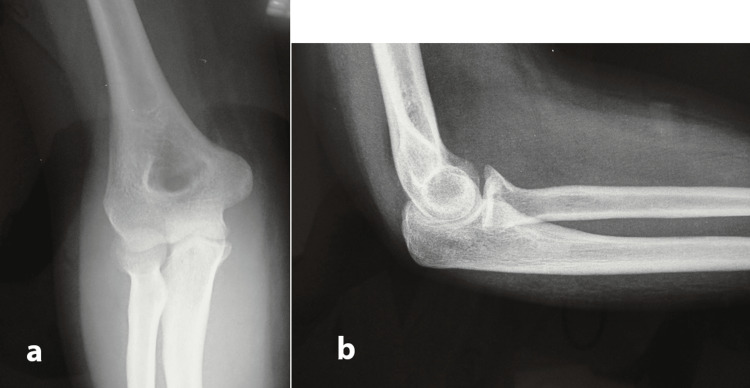
Three months post-injury anteroposterior (a) and lateral elbow (b) x-rays. Complete fracture healing noted on the coronoid process fracture.

A 23-year-old woman presented in the emergency department of our hospital reporting that she had a fall from a relatively small height. Imaging suggested a coronoid process fracture (type 2) with posterior elbow dislocation and associated subluxation of the DRUJ along with an intraarticular fracture of the distal radius, both on the dominant upper limb (Figure 1). The above injury was classified as an Essex-Lopresti injury with concomitant posterior elbow dislocation and distal radius intraarticular fracture, suggesting a more complex and rare variant of the original pattern. A local anesthetic was injected into the elbow joint and closed reduction was performed immediately (Figure 2). A CT scan was performed post-reduction to assess for intraarticular fragments, which turned negative. No motor or sensory neurologic deficits were found before or after reduction. The radial artery pulse and the capillary refill time were within normal limits, both before and after reduction. Due to the non-displaced nature of the fractures after reduction, we opted for conservative management. The upper extremity was immobilized with a long posterior splint, with the elbow at a 90-degree angle and the forearm in a neutral position. The patient was prescribed indomethacin in order to minimize the chances of heterotopic ossification. The split was removed after two weeks and with the assistance of a physiotherapist, progressive range of motion of both elbow and wrist joints was started. The physiotherapy was prescribed for three months. Three months later a radiologic evaluation showed complete fracture healing (Figure 3). One year post-injury the patient had achieved a complete range of motion regarding pronation, supination, and elbow flexion, with only a 10-degree deficit remaining.

## Discussion

Clinical diagnosis

Regarding the clinical evaluation of the injury, the suspicion should be set following a high-energy traumatic mechanism with an axial load on a supinated forearm [5]. Trousdale et al. found in 1992 that only 25% of ELI are diagnosed at presentation, although these numbers might have increased following the increasing literature reports of the injury [6]. Bike falls, road traffic accidents, falls by walking have been incriminated incidents [5]. If a radial head fracture is diagnosed, the IOM and DRUJ should be carefully assessed for instability and a high index of suspicion for the lesion is required in this case. Missed ELI can lead to devastating consequences, should they be treated on a chronic basis. The "C-Fingers comparative test" can be utilized to assess the IOM [7]. Intraoperatively, the radial-axial interosseous load (RAIL) test can provide valuable insight into the radioulnar stability and IOM integrity, provided that the radial head is not salvageable [8]. Ultrasound and MRI are also highly sensitive and considered the golden standard for diagnosing IOM lesions [9]. The Tilt test, the piano key sign, the radius pull test, the ulnocarpal stress test, and the ballottement test can be used to assess for DRUJ instability clinically, whereas intraoperative assessment can assist the final decision [5, 10]. Bilateral wrist X-rays are of high value to assess for proximal radius migration in comparison to the normal wrist and should be ordered after radial head fractures [10-11]. Proximal radial migration of more than 1.9mm should raise suspicion of IOM and ulnocarpal ligament complex and a forearm MRI should be ordered [11].

Therapeutic management

Previously, radial head excision was the intervention of choice, however, it is now widely accepted that this leads to proximal radial migration, positive ulnar variance, and devastating sequelae of events [1, 12-14]. Nowadays, this injury is approached according to the three lockers concept (PRUJ, IOM, DRUJ). Essex-Lopresti injury being a three-locker injury, the literature suggests that at least two lockers need to be restored to attain longitudinal stability [3, 12]. Numerous authors suggest that the PRUJ be restored by reconstruction or replacement of the radial head according to the Edwards Jupiter classification and that the DRUJ be pinned with K-wires [12, 15]. The Edwards and Jupiter classification is used as follows: Type 1: a large displaced radial head fractures that can be fixed using ORIF; Type 2: comminuted radial head fractures requiring radial head replacement; and Type 3: chronic ELI that can lead to proximal radial migration, persistent DRUJ instability and requires a personalized surgical approach to restore longitudinal stability [6, 15-16]. In the case of a radial head replacement, metal implants show superior outcomes compared to silicone [12]. Coronoid fractures can be seen in ELI variants such as our own and can be treated according to the Regan-Morrey classification, based on the fact that 50% of the coronoid process height needs to be maintained for elbow stability [17]. According to Regan and Morrey, there are three types of coronoid fractures: Type 1: avulsion of coronoid process tip; Type 2: fractures involving less than 50% of the coronoid process; and Type 3: fractures involving less than 50% of the coronoid process. In our case, the coronoid process fracture was categorized as type 2 according to the Regan-Morrey classification and was managed conservatively with reduction and immobilization. For the TFCC injury and DRUJ instability, K-wires can be utilized to immobilize the joint for one to two months, as the TFCC shows a healing capacity [12-14]. TFCC repair however might be needed in case of disruption [12-13]. The IOM tears on the other hand, although also managed conservatively, will not always respond well to immobilization due to their limited healing capacity [18]. Chronic ELI will leave functional deficits after treatment in up to 80% of patients [14, 16]. A variety of allograft and autograft procedures have been described for IOM reconstruction in chronic settings with non-consistent results [15]. Salvage operations such as ulnar osteotomy, Sauve-Kapandji procedure, and one bone forearm have been described on the ground of chronic complications [11-12].

The rarity of our case report lies in the conservative way of treatment followed. During our thorough literature review, only one case of conservative management was noted by Curr and Coe in 1946 [4, 19]. Our patient had an ELI with additional posterior elbow dislocation and distal radius intraarticular fracture and nevertheless responded great to the non-surgical management. This opens the discussion for a new way of treatment of these lesions. Conservative treatment has been utilized for the treatment of other high-energy elbow injuries as well, such as the floating elbow [20]. High suspicion should be held not only for the preliminary diagnosis of the injury, but also for the careful assessment of the three lockers, and, should enough stability be noted after reduction, forearm immobilization can be considered as a choice. In our patient, this management showed great results.

## Conclusions

Essex-Lopresti injury is an easy-to-miss diagnosis and a high level of suspicion should be maintained by the orthopedic surgeon. In the case of chronic cases, the results seem to be devastating. On radial head fractures, the wrists should be carefully examined, and should the clinical tests or wrist X-rays indicate DRUJ instability, we suggest that an MRI be performed. Surgical treatment is the current golden standard for most of these injuries. However, there is not an abundance of evidence regarding the treatment of Essex-Lopresti injuries and this case report shows that there is room for conservative management with excellent results.
